# Clinical Photography in Dermatology: A Cross-Sectional Analysis From the Patient’s Perspective in a Tertiary Care Center in Tamil Nadu, India

**DOI:** 10.7759/cureus.88112

**Published:** 2025-07-16

**Authors:** Aswath Surya, Dhanalakshmi K, Mohnish Sekar, Chinnu Preetham, Selva Sudha, Sudarvizhi A

**Affiliations:** 1 Department of Dermatology, Karpaga Vinayaga Institute of Medical Sciences and Research Center, Chengalpattu, IND

**Keywords:** clinical photography, dermatology, perception, skin lesion, smartphone

## Abstract

Introduction: Accurate diagnosis and therapy of skin illnesses depend significantly on visual inspection in dermatology, the medical specialty concerned with the diagnosis, treatment, and prevention of diseases of the skin, hair, nails, and mucous membranes. The study aims to identify the perception of patients toward clinical photography.

Materials and methods: This study uses a prevalidated, semistructured physical questionnaire with 23 questions, translated into the local language, to gather data from dermatology outpatient department patients. It focuses on patient demographics, experiences with medical photography, acceptability, and areas for improvement. Conducted at a tertiary care institute over six months, it employs a cross-sectional design with convenience sampling. The questionnaire addresses demographics, preferences, consent, and storage practices. Clinical photography, critical in dermatology, aids in diagnosis, treatment, education, and documentation through detailed visual records of skin conditions.

Results: The age distribution indicating that the largest group, 97/300 (32.3%), was over 60 years old, followed by those aged 18-30 years (54/300, 18%), 31-40 years (54/300, 18%), 51-60 years (52/300, 17.3%), and 41-50 years (43/300, 14.3%). The gender distribution was approximately equal, with women representing 151/300 (50.3%) and men 149/300 (49.7%) of the study population. Our study revealed that 157/300 (52.3%) of participants had prior experience with clinical photography, predominantly using smartphones, 106/157 (67.5%). Only 143/300 (47.7%) reported being asked for consent, with verbal consent being the most common, 84/143 (58.7%). Preferences favored department-owned equipment for photography and secure storage solutions, reflecting concerns about privacy. Participants expressed discomfort with publishing images in journals (30%) but showed greater acceptance for academic uses like sharing with other physicians 69/300 (23%). The need for improved awareness, detailed consent forms, and standardized practices was emphasized by the majority of participants.

Conclusion: The findings highlight the need for standardized consent protocols, transparent communication regarding the purpose and utilization of clinical photographs, and secure storage practices to maintain patient trust and confidentiality.

## Introduction

The accurate diagnosis and treatment of skin diseases heavily rely on visual inspection, which is a key aspect of dermatology, the medical specialty that focuses on these conditions [1]. For an extended period, dermatologists have recorded their patients' skin disorders through descriptive notes and clinical expertise [2]. Conversely, clinical photography has transformed dermatological practice by offering a comprehensive, standardized, and reproducible method for documenting skin lesions [3]. The application of black-and-white film photography for documenting dermatological diseases dates back to the early 1900s, marking the inception of clinical photography within the field of dermatology [4]. The advent of digital imaging systems, high-resolution cameras, and specialized imaging modalities such as dermoscopy and reflectance confocal microscopy has significantly altered the landscape of clinical photography. Technological advancements have enhanced the clarity, quality, and diagnostic utility of clinical images, enabling dermatologists to detect even the most subtle changes in skin with exceptional accuracy [5,6].

As a visual record of skin lesions at different phases of diagnosis and therapy, clinical photography is an essential tool for documenting dermatological diseases [7]. Accurate diagnosis and differential diagnosis are made possible by high-quality clinical photographs that dermatologists may use to study skin lesions in detail, including their shape, location, and progression [8]. Moreover, sequential imaging can track complications, treatment efficacy, and disease progression. A thorough understanding of the patient's initial response and response to therapy is essential for accurate treatment planning in dermatology. Importantly, dermatologists can get objective proof of disease severity, therapy success, and adverse effects through clinical photography [9]. By comparing photographs taken at the beginning and end of a treatment plan, dermatologists can better meet the needs of their patients while reducing the likelihood of unwanted side effects.

Clinical photography is a great resource for dermatology residents, medical students, and dermatologists in their training. These photographs help in learning how to diagnose, recognize patterns, and make differential diagnoses by capturing a wide variety of dermatological disorders in real-life clinical situations. To further improve learning and understanding, clinical photos are also used in instructional materials, textbooks, and internet tools.

Giving patients the tools they need to be an active part of their treatment is essential. Dermatologists can use clinical photography to visually explain disease processes, treatment options, and self-care practices to their patients. Patients can better understand their condition, treatment goals, and progress by looking at their own clinical images, which encourages them to stick to their treatment plans and participate in decision-making. There are several ethical concerns that arise when using clinical photography in dermatology, including questions of patient confidentiality, permission, and privacy, despite the many benefits of the practice. To ensure that patients are aware of the purpose, hazards, and possible uses of their pictures, dermatologists must get informed consent before taking clinical images. Data protection requirements require additional security procedures, such as the deidentification of photos, encryption of data, and the safeguarding of storage devices, to ensure patient confidentiality [7-9].

In Europe, organizations like the Institute of Medical Illustrators have developed and implemented standards for medical photography within clinical environments [10-13]. Historically, patient feedback has often been overlooked in the development of recommendations. A deficiency exists in the published data from our country regarding patients' perspectives on clinical photography. In patient treatment, it is essential for physicians to exercise caution in the acquisition, storage, and dissemination of clinical images to prevent potential violations of patient privacy and ethical standards. This study aims to enhance photography techniques within the dermatology community by examining patients' preferences and concerns related to clinical photography from a comprehensive viewpoint. While clinical photography is increasingly used in dermatology worldwide, there are limited data from India exploring patients' awareness, comfort, and consent-related concerns. This study addresses a critical gap by examining patient perspectives within the sociocultural context of a tertiary care center in Tamil Nadu.

Objectives

This study aimed to explore how patients perceive consent, privacy, and the use of clinical photography in dermatology in a tertiary care setting in India, and to assess the patient preferences and comfort levels regarding equipment, personnel, consent, and usage of clinical photographs in dermatological practice.

## Materials and methods

This research employs a validated, semistructured questionnaire consisting of 23 items, translated into the local language, to collect data from patients. Expert validation was used. The responses were recorded and analyzed, concentrating on four primary domains: patient demographic characteristics, prior experiences with medical photography, acceptability and preferences, and potential areas for improvement.

This research employs a cross-sectional design conducted at a tertiary care center in Tamil Nadu over a period of six months. The study population comprises patients visiting the dermatology outpatient department (OPD) of the institute.

The inclusion criteria consist of patients aged 18 years and older, possessing a minimum of primary education, who are visiting the dermatology OPD and are willing to participate. Individuals who are unwilling to participate in the study are excluded from consideration. The sampling frame includes patients aged 18 years and older who present to the dermatology OPD of a tertiary care hospital in Tamil Nadu, India.

Convenience sampling was employed, and the sample size of 300 was considered for this study. Convenience sampling was used due to practical feasibility, as the study was conducted in a tertiary care dermatology outpatient setting with limited resources and time. This method allowed us to recruit participants who were readily available and willing during their routine clinic visits, making data collection more efficient. While it may limit generalizability, it was appropriate for exploring preliminary insights into patient perspectives in our specific context. The main study instrument is a 23-item prevalidated semistructured questionnaire based on the work by AlSuhaymi et al. [14] shown in the Appendix, which will undergo modification, translation into the local language, and validation. The questionnaire encompasses patient demographic characteristics (questions 1-4), prior experiences with medical photography and preferences (questions 5-11), consent and storage of medical images (questions 12-21), and recommendations for enhancement (questions 22 and 23). The process of data collection entails the administration of the translated questionnaire to patients, followed by the analysis of responses to ascertain perspectives and preferences. Ethical approval was obtained from the institutional ethical committee of Karpaga Vinayaga Institute of Medical Sciences and Research Center (KIMS/PG/15/20.09.2024).

Statistical analysis

Descriptive statistics were reported as mean (SD) for continuous variables and frequencies (percentage) for categorical variables. Data were statistically evaluated with IBM Statistical Package for the Social Sciences Statistics for Windows, version 26.0 (IBM Corp., Chicago, IL).

## Results

The study comprised 300 participants, with the age distribution indicating that the largest group, 97/300 (32.3%), was over 60 years old, followed by those aged 18-30 years (54/300, 18%), 31-40 years (54/300, 18%), 51-60 years (52/300, 17.3%), and 41-50 years (43/300, 14.3%). The gender distribution was approximately equal, with women representing 151/300 (50.3%) and men 149/300 (49.7%) of the study population. The majority of participants were individuals with primary education, 164/300 (54.7%); undergraduate education, 70/300 (23.3%); and higher secondary education, 66/300 (22%). Employment status revealed that employed individuals and students each comprised 102/300 (34%) of the participants, whereas 96/300 (32%) were unemployed. Table 1 shows all the demographic details.

**Table 1:** Distribution of demographic variables among the study participants (n = 300)

Sl. no.	Variable	Category	Frequency	Percentage
1	Age (years)	18-30	54	18%
		31-40	54	18%
		41-50	43	14.3%
		51-60	52	17.3%
		>60	97	32.3%
2	Gender	Female	151	50.3%
		Male	149	49.7%
3	Educational status	Undergraduate	70	23.3%
		Higher secondary	66	22%
		Primary	164	54.7%
4	Employment status	Employed	102	34%
		Student	102	34%
		Unemployed	96	32%

The study assessed the preferences and perspectives of 300 participants regarding clinical photography (Table 2). Just over half (52.3%) had prior experience with clinical photographs, with smartphones being the most commonly used device (67.5%). Institutional cameras and personal equipment were equally preferred (27.7% and 25%, respectively). Comfort levels were nearly evenly split, with 51.7% comfortable and 48.3% uncomfortable. Regarding preferred photographers, 36% chose physicians, followed by nurses (33.7%) and nonmedical staff (30.3%). Gender preferences varied, with 36.3% preferring the opposite gender, 29.7% the same, and 34% having no preference. Responses showed mixed views on photography’s usefulness, with 28.7% stating that it clarified doubts, 34% reporting a better understanding, and 37.3% remaining neutral. These findings highlight the need for patient-centered approaches in clinical photography practices.

**Table 2:** Distribution of preferences and perspectives on clinical photography among the study participants (n = 300)

Sl. no.	Variable	Category	Frequency	Percentage
1	Clinical picture taken before	Yes	157	52.3%
		No	143	47.7%
2	Type of camera used (n = 157)	Digital	49	31.2%
		Smart phone	106	67.5%
3	Preferred equipment for clinical photographs	Dermatologist personal	83	27.7%
		Dermatologist phone	70	23.3%
		Institution camera	75	25%
		Institution phone	72	24%
4	Comfortable with clinical photography	Yes	155	51.7%
		No	145	48.3%
5	Preferred person for taking photographs	Physician	108	36%
		Nurse	101	33.7%
		Nonmedical staff	91	30.3%
6	Preferred gender of photographer	Member of the opposite gender	109	36.3%
		Member of the same gender	89	29.7%
		No preference	102	34%
7	Feedback on questionnaire	Helped clarify doubts	86	28.7%
		Improved understanding	102	34%
		No opinion	112	37.3%

The study explored participants' views on clinical photography practices and consent (Table 3). Just over half (51.3%) supported the need for photographers to wear an authorized badge. Only 47.7% reported that consent was obtained before photography, with verbal consent being the most common (58.7%), followed by written (31.5%) and video (11.8%). While 52% believed consent should be mandatory, 48% did not. Among those who withdrew consent, 35% felt ignored, while 33.1% did not share that sentiment. These findings emphasize the need for clear consent protocols and greater sensitivity to patient perceptions to ensure ethical clinical photography practices.

**Table 3:** Distribution of responses on clinical photography practices and consent among the study participants (n = 300)

Sl. no.	Variable	Category	Frequency	Percentage
1	Authorized badge for a photographer	Yes	154	51.3%
		No	146	48.7%
2	Consent asked for a photograph	Yes	143	47.7%
		No	157	52.3%
3	Type of consent	Verbal consent	84	58.7%
		Video consent	17	11.8%
		Written consent	42	29.4%
4	Belief in mandatory consent	Yes	156	52%
		No	144	48%
5	Felt overlooked after declining consent	Yes	105	66.9%
		No	52	33.1%

The study assessed participants' perspectives and suggestions regarding the use and management of clinical photographs (Table 4). The most preferred storage method was the physician’s personal phone (29%), followed by institutional devices and files. While 53% emphasized the need for secure storage, 47% did not. Postconsultation preferences varied, with 34.3% wanting a copy of their image, 33.3% preferring to view it later, and 32.3% having no preference. Only 46% reported that the purpose of photography was explained to them. Acceptable uses for unidentifiable images included sharing with treating physicians (23%), medical education (22%), publications (18.7%), personal storage (18.3%), and academic sharing via WhatsApp (18%). A majority (55.3%) believed photography purposes should be clearly stated in consent. Suggestions for improvement included raising awareness (37.3%) and providing detailed written consent (30.7%), while 32% had no specific recommendation. These findings underscore the importance of transparency, secure practices, and patient education in clinical photography.

**Table 4:** Distribution of participant opinions and suggestions on clinical photography and its utilization among the study participants (n = 300)

Sl. no.	Variable	Category	Frequency	Percentage
1	Preferred storage of pictures	In the institution/clinic files	69	23%
		In the institution/clinic phone	76	25.3%
		In the physician’s personal computer	68	22.7%
		In the physician's personal phone	87	29%
2	Storage security requirement	Yes	159	53%
		No	141	47%
3	Preference after consultation	Get a copy of your picture	103	34.3%
		No preference	97	32.3%
		See the picture after consultation/stay in the hospital	100	33.3%
4	Purpose of photography explained	Yes	138	46%
		No	162	54%
5	Acceptable use of unidentifiable pictures	Publishing in medical journals	56	18.7%
		Sharing via WhatsApp for academic purposes	54	18%
		Sharing with other physicians involved	69	23%
		Storing in personal collection	55	18.3%
		Using for the education of medical students	66	22%
6	Should purposes be stated in consent	Yes	166	55.3%
		No	134	44.7%
7	Suggestions for improvement	No opinion	96	32%
		More detailed written consent form	92	30.7%
		More information and awareness about utilization	112	37.3%

## Discussion

Our study investigated patient perspectives on clinical photography within a dermatology outpatient setting, focusing on prior experiences, consent practices, and preferences regarding the process. A slight majority of participants, 157/300 (52.3%), reported having had clinical photographs taken previously, with smartphones being the predominant device used, 106/157 (67.5%). This aligns with the increasing integration of mobile devices in medical settings due to their convenience and accessibility. This finding aligns with trends reported by AlSuhaymi et al., where only 105/414 (25.4%) of participants had prior experience with clinical photography, and camera phones were used in 47/414 (11.4%) of cases compared to 55/414 (13.3%) for digital cameras [14]. The acceptance of physician-owned professional cameras, 115/300 (38.4%), was higher in our population compared to the study by Hacard et al., 53/198 (26.8%) [13].

Approximately half of the participants, 155/300 (51.7%), expressed comfort with clinical photography. Preferences for the individual taking the photographs varied, with 108/300 (36%) favoring physicians, 101/300 (33.7%) preferring nurses, and 91/300 (30.3%) indicating no preference. Physician photographers were the most accepted choice in our study (108/300, 36%), consistent with AlSuhaymi et al. [14], where 344/414 (83%) of participants preferred physicians. Similarly, Hacard et al. reported that patients prefer physicians to act as photographers, reflecting the trust they place in their primary healthcare providers (180/198, 91%) [13]. This sentiment was echoed by AlSuhaymi et al., where 312/414 (54.8%) agreed that medical photography is comparable to other medical procedures like X-rays or CT scans [14].

Gender preferences were also noted, with 109/300 (36.3%) favoring a photographer of the opposite gender, 89/300 (29.7%) preferring the same gender, and 102/300 (34%) expressing no preference. These findings are consistent with studies by Hacard et al. [13], in which patients indicated that the gender of the photographer did not matter (119/198, 59.9%), and with AlSuhaymi et al., who found that 246/414 (59.4%) of participants expressed no gender preference [14]. These findings underscore the importance of accommodating patient preferences to enhance comfort and trust during the clinical photography process.

Notably, only 143/300 (47.7%) of participants reported being asked for consent before photographs were taken, highlighting a significant gap in practice. Among those who provided consent (n = 143), verbal consent was the most common form, 84/143 (58.7%), followed by written consent, 42/143 (29.4%), and video consent, 17/143 (11.8%). A slight majority, 156/300 (52%), of participants did not believe that obtaining consent was mandatory, and 162/300 (54%) reported that the purpose of the photography was not explained to them. This indicates a need for better communication regarding the reasons for clinical photography and the importance of informed consent. Comparatively, AlSuhaymi et al. found that verbal consent was more commonly sought (81/414, 19.6%) than written consent (13/414, 3.1%), though 307/414 (74.2%) reported no consent being taken because they were not photographed [14].

Our findings reveal a significantly lower rate of consent acquisition (only 47.7%) and awareness regarding the purpose of clinical photography (only 46%) compared to global studies, where higher standards of informed consent and patient communication are often reported. This discrepancy highlights a critical gap in current dermatological practice in India, where standardized protocols and patient education may be lacking. The novelty of our study lies in bringing attention to these underresearched patient perspectives in the Indian healthcare setting, underlining the urgent need for ethical guidelines and awareness campaigns tailored to local contexts.

Regarding the storage of clinical photographs, 87/300 (29%) preferred storage on the physician's personal phone, followed by the institution's phone, 76/300 (25.3%), institution/clinic files, 69/300 (23%), and the physician's personal computer, 68/300 (22.7%). This distribution reflects varying levels of trust in different storage methods. Additionally, 159/300 (53%) of participants expressed concerns about storage security, underscoring the importance of robust data protection measures. Publishing unidentifiable images in medical journals was acceptable to only 56/300 (18.7%), significantly lower than the acceptance rates reported among British patients (218/250, 87%) and Chinese patients (260/280, 92.8%) [15,16]. In our study, only 138/300 (46%) reported that the purpose of photography was explained, compared to 373/414 (90.1%) in AlSuhaymi et al., who believed all utilizations of photographs should be explicitly stated in the consent form. Interestingly, participants in AlSuhaymi et al. were more willing to accept photographs for academic purposes, such as sharing with other physicians (84/414, 20.2%) or for medical student education (56/414, 13.5%), compared to the relatively low acceptability of publication in medical journals (28/414, 6.7%) [14].

Participants indicated varying levels of acceptance for the use of unidentifiable images, with 69/300 (23%) approving sharing with other physicians involved in their management, 66/300 (22%) supporting use for medical student education, and 56/300 (18.7%) agreeing to publication in medical journals. This suggests that patients are generally supportive of using their images for educational and professional purposes, provided their identity is protected. Comparatively, AlSuhaymi et al. reported higher acceptance of storage in department archives (150/414, 36.2%) and password-protected computers (116/414, 28.0%), highlighting the importance of secure storage systems to address patient concerns about data misuse.

When asked for suggestions to improve the clinical photography process, 112/300 (37.3%) of participants called for more information and awareness about the utilization of clinical photographs, while 92/300 (30.7%) recommended a more detailed written consent form. This feedback highlights the need for enhanced patient education and more comprehensive consent procedures to ensure patients are fully informed and comfortable with the process. Similarly, AlSuhaymi et al. reported that 166/414 (40.2%) of participants sought greater awareness and information, and 111/414 (26.7%) suggested more detailed written consent forms [14]. These findings highlight the need for improved communication and standardized consent pathways. The study’s limitations include the use of convenience sampling and a single-center setting, limiting generalizability. Additionally, reliance on self-reported data introduces potential biases.

## Conclusions

This cross-sectional study provides valuable insights into patients' perspectives on clinical photography in dermatology within a tertiary care setting in Tamil Nadu. The findings underscore a generally positive attitude among patients toward the use of clinical photography, particularly when clear communication, informed consent, and privacy safeguards are maintained. However, concerns regarding confidentiality, the purpose of image usage, and the preference for nonidentifiable images highlight the need for standardized protocols and ethical practices. Enhancing patient awareness and ensuring transparent practices can foster greater trust and acceptance, ultimately improving the quality of dermatological care and medical documentation. Future initiatives should focus on developing patient-centered guidelines and integrating digital tools that prioritize ethical considerations in clinical photography.

## Appendices

Questionnaire

**Table 5:** Questionnaire

Sl. no.	Question	Options
1	What is your age?	☐ 18-30 years ☐ 31-40 years ☐ 41-50 years ☐ 51-60 years ☐ Above 60 years
2	What is your gender?	☐ Male ☐ Female
3	What is your highest level of education?	☐ Primary ☐ Higher secondary ☐ Undergraduate
4	What is your current employment status?	☐ Employed ☐ Student ☐ Unemployed
5	Have you ever had a clinical photograph taken at a dermatology institution or clinic?	☐ Yes ☐ No
6	If yes, what type of camera was used?	☐ Digital camera ☐ Smartphone camera
7	What type of equipment do you prefer the dermatologist to use for clinical photographs?	☐ Institutional camera ☐ Institutional phone ☐ Dermatologist’s personal camera ☐ Dermatologist’s personal phone
8	Are you comfortable with being clinically photographed?	☐ Yes ☐ No
9	Who do you prefer to take your clinical photograph?	☐ Physician ☐ Nurse ☐ Nonmedical staff
10	What is your preference for the gender of the person taking the clinical photograph?	☐ Member of the same gender ☐ Member of the opposite gender ☐ No preference
11	Do you believe it is necessary for the person taking the clinical photograph to wear an authorized identification badge?	☐ Yes ☐ No
12	Were you asked for your consent before the clinical picture was taken? (if applicable)	☐ Yes ☐ No
13	If yes, what type of consent was obtained?	☐ Verbal consent ☐ Written consent ☐ Video consent
14	Do you think consent in any form is necessary before taking clinical photographs?	☐ Yes ☐ No
15	Have you ever felt overlooked by a dermatologist after declining to give consent for clinical photography? (if applicable)	☐ Yes ☐ No
16	Where do you find it acceptable to store clinical photographs?	☐ Institution/clinic files ☐ Institution/clinic phone ☐ Physician’s personal computer ☐ Physician’s personal phone
17	Do you think the storage location of your photograph should be password-protected and encrypted?	☐ Yes ☐ No
18	At the end of the consultation, what is your preference?	☐ See the picture after consultation/stay in hospital ☐ Get a copy of your picture ☐ No preference
19	Was the purpose of taking the photograph clearly explained to you? (if applicable)	☐ Yes ☐ No
20	Do you find it acceptable to use deidentified photographs for the following purposes?	☐ Publishing in medical journals ☐ Sharing with other physicians ☐ Medical student education ☐ WhatsApp academic sharing ☐ Storing in personal collection
21	Do you think all purposes of photograph use should be stated in the consent form?	☐ Yes ☐ No
22	How can medical photography in dermatology be improved?	☐ More information and awareness ☐ Detailed written consent form ☐ No opinion
23	How did this questionnaire help you?	☐ Helped clarify doubts ☐ Improved understanding ☐ No opinion
